# Straightened Small Pinnae in *TRPV4* c.1024G>T Heterozygous Cats

**DOI:** 10.1002/age.70114

**Published:** 2026-05-06

**Authors:** Yuki Matsumoto, Ryuga Ishii, Hisashi Ukawa, Saaya Hiyoshi‐Kanemoto, Hinako Hayashi, Haruka Onishi, Kai Ataka, Ryo Horie

**Affiliations:** ^1^ Genetic Testing Section Anicom Pafe Inc Yokohamashi‐Nakaku Kanagawa Japan; ^2^ Research and Development Section Anicom Specialty Medical Institute Inc Yokohamashi‐Nakaku Kanagawa Japan; ^3^ Data Science Center Azabu University Sagamihara‐shi Kanagawa Japan

**Keywords:** domestic cat, fold ear, Scottish folds, *TRPV4*

## Abstract

The folded‐ear phenotype of Scottish Fold cats results from a dominant variant of the *TRPV4* gene (c.1024G>T). Producing homozygous individuals is discouraged due to severe osteochondrodysplasia and identifying heterozygous carriers is critical for breeding. Although carriers are generally expected to have folded ears, straight‐eared individuals have also been suspected among them. Here, we investigated the relationship between ear phenotype and *TRPV4* genotype using longitudinal photographic data and *TRPV4* genotyping in 114 cats. We identified seven individuals that transitioned from folded to straight ear phenotype during maturation. Genotyping confirmed all were heterozygous carriers, with 12.7% found among heterozygous cats. Morphometric analysis of 14 cats with high‐quality photographs demonstrated these “straightened” cats had smaller pinnae than genetically wild‐type straight‐eared cats (*p* < 0.05), suggesting cartilage anomalies. These findings confirm “cryptic folds”—phenotypically straight but genetically heterozygous cats. Consequently, visual inspection for breeding selection is insufficient, highlighting the necessity of *TRPV4* genotyping to prevent unintentional production of homozygous offspring with severe osteochondrodysplasia.

1

The characteristic folded‐ear phenotype in Scottish Fold cats was previously found to show incomplete dominance (Takanosu et al. 2008). The causative missense variant in *TRPV4* (c.1024G>T) was subsequently identified and has been implicated in auricular cartilage malformation (Gandolfi et al. 2016). Homozygous individuals (T/T) develop severe Scottish Fold osteochondrodysplasia, a progressive skeletal disorder, and their breeding is discouraged due to welfare concerns. Accurate identification of the variant allele is critical for breeding management. Heterozygous cats (G/T) exhibit varying degrees of skeletal abnormalities (Takanosu and Hattori 2020; Rorden et al. 2021; Sartore et al. 2023). Typically, heterozygous cats exhibit folded ears, while wild‐type individuals (G/G) possess straight ears (Scottish Straight). These phenotypes are completely linked to their respective genotypes in the previous study (Anderson et al. 2022), with limited literature describing straight ears in heterozygous individuals. However, breeders and veterinarians have observed cats with straight ears carrying the genetic factor for fold ears (Vella et al. 1999). If *TRPV4* carriers have straight ears, visual selection may fail to identify carriers, leading to the unintentional use of “cryptic folds” in breeding and increased risk of homozygous offspring. To date, this phenotypic plasticity remains unvalidated using phenotypic records and genotypic data. This study aimed to investigate the relationship between ear phenotype and *TRPV4* genotype in a Scottish Fold population using longitudinal photographic data and DNA samples from a pet insurance company. We determined the genotype of individuals that change from folded to straight ears and characterized their ear phenotype through relative size measurements.

In 2022, owners of 18,729 cats registered as Scottish Folds and insured with Anicom Insurance Inc. were recruited to participate in this study. Oral mucosal tissue was collected via buccal swabs from participating cats, and DNA was extracted using the Chemagic DNA Buccal Swab Kit (PerkinElmer, MA, USA) or DNAdvance Kit (Beckman Coulter, CA, USA). A custom Illumina Infinium XT iSelect 96 kit (Illumina, CA, USA) was used for genotyping of a *TRPV4* variant (c.1024G>T, OMIA 000319–9685). Sanger sequencing confirmed ambiguous markers using KOD‐Plus v.2 polymerase (TOYOBO, Osaka, Japan) with the following primers: Forward: ACCAGCCCCACATCGTC; Reverse: CCCAATCTTGCCGGTCTTGGCGGCCATC. Sequencing used BigDye Terminator v3.1 (Thermo Fisher Scientific, MA, USA), analyzed on a 3730xl DNA Analyzer (Thermo Fisher Scientific). Target amplification of *TRPV4* was performed using Felis_catus_9.0 as reference.

Phenotypic classification and morphometry based on longitudinal photographic data was performed. Photographs collected at the time of insurance enrollment and subsequent annual renewals were used. Enrollment images captured kittens (3–11 months), whereas subsequent annual updates showed adult cats (1 year or older). Photographs were excluded if ear traits were unclear. Ear phenotypes (folded or straight) were assessed by two independent observers. Only individuals with matching observer classification were analyzed. “Straight ears” were defined as straight ears without forward folding. We used two datasets: Dataset 1 included at least two photographs for each cat, and Dataset 2 included those cats from Dataset 1 that also had measured ear lengths. Dataset 1 was restricted to individuals with continuous contract history of at least 2 years, including kitten and adult stages. Cats were classified into three groups: Fold‐Fold group (F‐F): Folded ears at both stages. Straight‐Straight group (S‐S): Straight ears at both stages. Fold‐Straight group (F‐S): Folded ears at kitten stage but straight ears at adult stage (Figure 1a). Genotype frequency differences between sexes were assessed using the chi‐square test. In addition, sex‐based differences in phenotypic straightening among heterozygous cats were assessed using Fisher's exact test.

**FIGURE 1:**
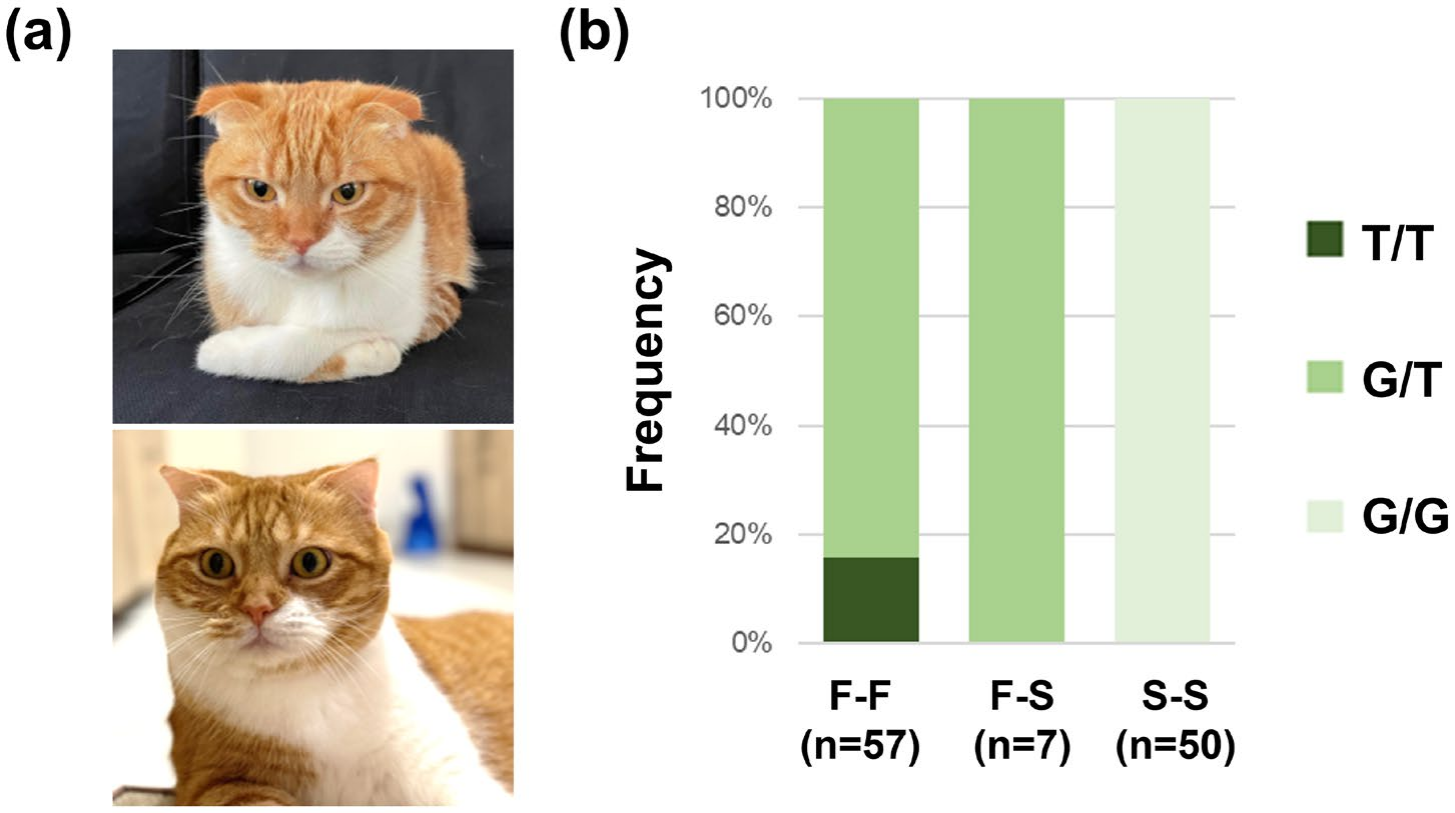
Relationship between *TRPV4* (c.1024G>T) genotype and ear morphology. (a) A case of phenotypic straightening in a Fold‐Straight. (b) *TRPV4* variant frequency by three ear types.

We calculated the Relative Ear proportion Ratio (RER) to quantify pinna size relative to face size for S‐S and F‐S groups from Dataset 2. The F‐F group was excluded due to difficulty measuring pinna size from photographs. Dataset 2 includes frontal facial photographs. The length of width and height for pinna and face were measured using ImageJ 1.54 g software (National Institute of Health, Maryland, USA). The RER was derived by dividing bilateral pinna areas (height multiplied by width) by facial area (maximum facial width multiplied by facial height) (Figure 2a). The lines representing height were perpendicular to lines representing width (at pinna base and the center of the face). Differences in RER between the two groups were analyzed using the Wilcoxon rank sum test. All statistical analyses were performed using R software v4.4.1.

**FIGURE 2:**
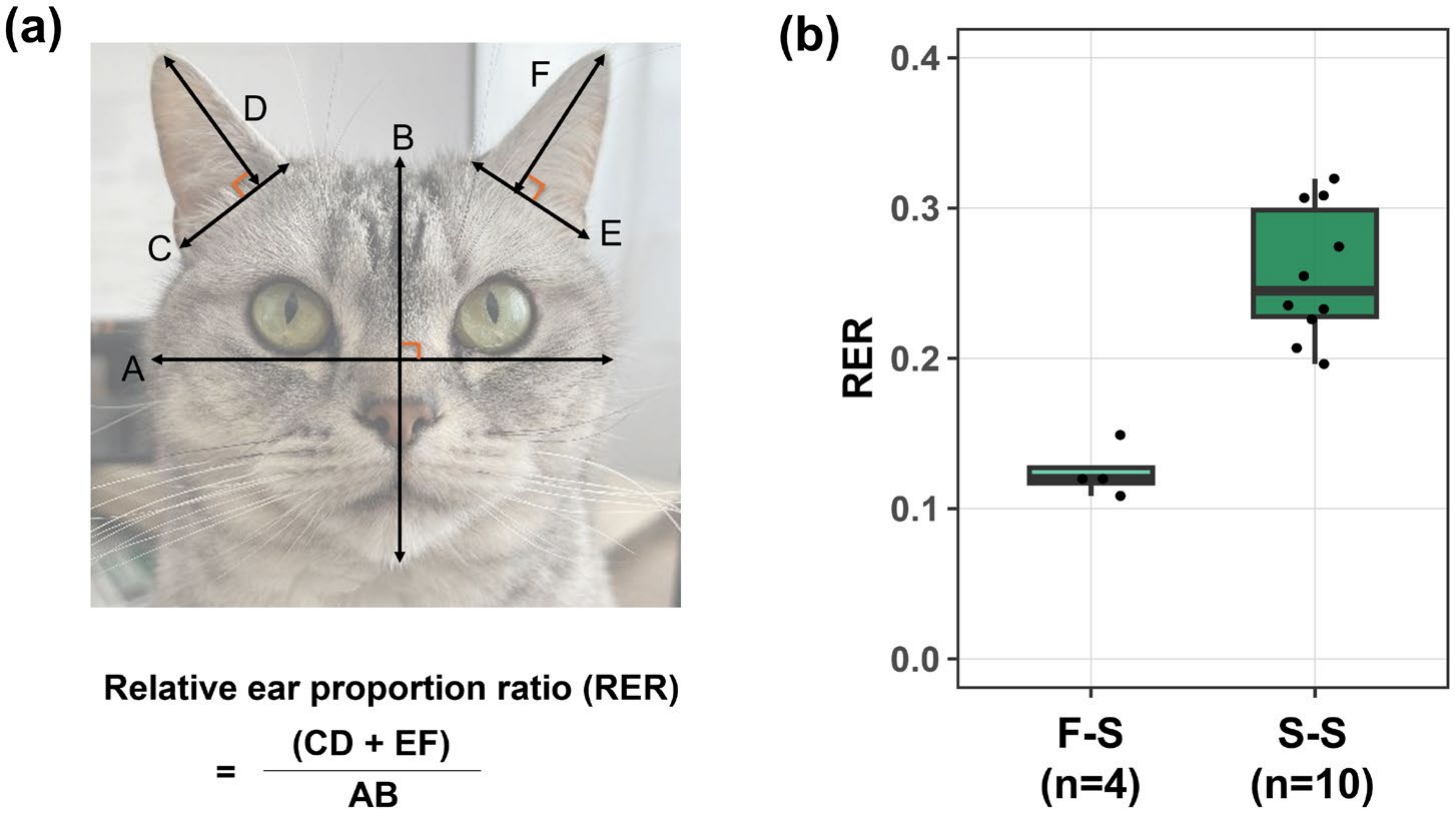
Phenotypic straightening and small pinnae in *TRPV4* c.1024G>T variant heterozygous cats. (a) Schematic representation for calculating the Relative Ear Proportion Ratio (RER). (b) Relative pinnae size comparison by two groups.

Of 18 729 cats initially recruited, samples were collected from 418 individuals. After screening for photographic quality, a total of 114 cats were retained for Dataset 1. Based on ear phenotype, these cats were classified into three groups: 57 in the fold‐fold group (F‐F), 7 in the fold‐straight group (F‐S), and 50 in the straight‐straight group (S‐S). The genotype distribution in Dataset 1 was as follows: 9 homozygous (T/T), 55 heterozygous (G/T), and 50 wild‐type (G/G) individuals. Across phenotype groups, the F‐F group included 48 heterozygotes and 9 homozygotes, the F‐S group included 7 heterozygotes, and the S‐S group included 50 wild‐type cats. Notably, 7 of the 55 heterozygous cats (12.7%) showed phenotypic straightening of the pinna (F‐S group; Figure 1a,b). We define these cats as cryptic folds, namely cats that are phenotypically straight‐eared but genetically heterozygous for the *TRPV4* c.1024G>T variant. These results show that adult straight‐eared cats include both true wild‐type individuals and cryptic folds. No significant difference was observed in *TRPV4* genotype distribution between males and females (*p* > 0.05, chi‐square test). Among 55 heterozygous individuals, phenotypic straightening was observed in 10.0% (3/30) of males and 16.0% (4/25) of females, showing no significant difference (*p* > 0.05, Fisher's exact test), suggesting sex does not influence phenotypic straightening likelihood. Morphometric analysis comparison of RER for 14 cats from Dataset 2 indicated that F‐S cats exhibited significantly smaller pinnae than wild‐type S‐S cats, with a median value approximately half that of the S‐S group (median: 0.12 (F‐S group) vs. 0.25 (S‐S group); *p* < 0.05; Figure 2b).

Even before the causative mutation was identified, breeders and veterinarians had observed that cats with straight ears can carry a genetic factor for fold ears (Vella et al. 1999). Our findings indicate that this occurs at ~12.7% frequency in our cohort, having implications for breeding management. Breeding pairs that can produce *TRPV4* homozygous offspring (i.e., Fold‐to‐Fold matings) should be strictly avoided, as homozygous individuals suffer from severe osteochondrodysplasia (Gandolfi et al. 2016; Takanosu and Hattori 2020). Standard mating of “folded” with “straight” cats assumes straight‐eared cats are genetically wild‐type. However, if a breeder misidentifies a “cryptic fold” (an F‐S group) as a Scottish Straight for breeding, this could result in a “heterozygote × heterozygote” mating, risking offspring with severe homozygous osteochondrodysplasia.

The severity and progression of osteochondrodysplasia vary among *TRPV4* heterozygous cats (Gandolfi et al. 2016; Rorden et al. 2021; Sartore et al. 2023). Our data indicate that the structural integrity of the pinna may increase with growth to support straight pinnae in some heterozygous cats. However, the smaller relative pinna size in the F‐S group suggests cartilage anomalies. Although the molecular mechanisms distinguishing straightened (F‐S) from persistently folded ears (F‐F) remain unclear, the restriction of this phenomenon to heterozygotes suggests involvement of the *TRPV4* gene, linked variants, or epigenetic factors. Supplementary owner questionnaires for five F‐S individuals revealed four developed straight ears by 1 year of age. One individual exhibited seasonal fluctuation—folded ears in winter, straight ears in summer—before establishing a fixed straight phenotype by 3 years of age. Although temperature‐dependent ear morphology in cats has not been documented, it is noteworthy that *TRPV4* encodes a thermosensitive ion channel activated by warm temperatures (Watanabe et al. 2002; Shibasaki 2020). This raises the possibility of environmental heat modulating *TRPV4* activity and contributing to seasonal variation in cartilage stiffness; however, this interpretation is based on a single individual and should be regarded as preliminary. These observations suggest that, in addition to genetic factors, aging, and potentially environmental temperature, may contribute to phenotypic variability in a subset of cases, warranting further investigation. Notably, studies characterizing ear phenotypes with genotype data in cats of Western origin found that all *TRPV4* variant heterozygous cats exhibited folded ears (Rorden et al. 2021; Anderson et al. 2022; Sartore et al. 2023), in contrast to the 12.7% straightening frequency observed in our cohort. This discrepancy might stem from variations in age at assessment and/or environmental factors, such as ambient temperature, as well as genetic background differences across populations (Matsumoto et al. 2021).

Although our findings provide insights into the “cryptic fold” phenomenon, several limitations should be noted. First, phenotypic assessment relied on two‐dimensional photographs, which precluded the evaluation of cartilage structure and stiffness through palpation, along with clinical records. No phenotypic comparison for the F‐F group was assessed. The correlation between phenotypic straightening and systemic osteochondrodysplasia severity remains undetermined. Second, the sample size of the F‐S group was limited (*n* = 7), preventing robust statistical analysis. Moreover, the relatively small sample size of heterozygous cats (*n* = 55) may limit the generalizability of the 12.7% straightening frequency. Further validation in a larger, independent cohort is needed to confirm this estimate. Future studies using whole‐exome sequencing and/or epigenetic analysis including environmental data are warranted to elucidate this phenotypic variability.

In conclusion, a straight‐eared phenotype in adult Scottish Folds does not guarantee a wild‐type *TRPV4* genotype. Although “cryptic folds” can be distinguished through measurements of their smaller size, they are indistinguishable by visual inspection alone. Therefore, *TRPV4* genetic testing is essential to identify carriers and prevent homozygous breeding, enhancing feline welfare.

## Author Contributions

**Yuki Matsumoto:** conceptualization; data curation; formal analysis; supervision; visualization; writing – original draft; writing – review and editing. **Ryuga Ishii:** data curation; writing – original draft; formal analysis; writing – review and editing. **Hisashi Ukawa:** data curation; formal analysis; visualization; writing – review and editing. **Saaya Hiyoshi‐Kanemoto:** supervision; data curation; writing – original draft; writing – review and editing. **Hinako Hayashi:** supervision; writing – review and editing. **Haruka Onishi:** supervision; writing – review and editing. **Kai Ataka:** supervision; writing – review and editing. **Ryo Horie:** supervision; writing – review and editing.

## Funding

This work was supported by the Research Foundation of the Anicom Specialty Medical Institute Inc.

## Ethics Statement

All procedures were conducted in accordance with institutional, national, and international ethical guidelines. All swab samples were obtained with the consent of the owners. This study was approved by the Ethics Committee of Anicom Specialty Medical Institute Inc. (ID: 2024‐01).

## Consent

All authors have read and approved the final version of this manuscript.

## Conflicts of Interest

Y.M. is an employee of Anicom Pafe Inc., a DNA testing company that offers commercial testing for the variant described in this study, and Anicom Specialty Medical Institute Inc., a sister company of Anicom Pafe Inc. H.U., H.O., and K.A. are employees of Anicom Pafe Inc. S.H.‐K., R.I., and R.H. are employees of Anicom Specialty Medical Institute Inc.

## Data Availability

Data sharing not applicable to this article as no datasets were generated or analysed during the current study.
